# Bone union achieved in an ulnar shaft nonunion using dextrose prolotherapy without surgery: a case report

**DOI:** 10.1093/jscr/rjaf741

**Published:** 2025-09-15

**Authors:** Shinnosuke Hada, Kengo Sugitani, Hiroaki Kanazawa, Muneaki Ishijima

**Affiliations:** Department of Orthopaedic Surgery, Juntendo University, Tokyo Koto Geriatric Medical Center, 3-3-20 Shin-Suna, Koto-ku, Tokyo 136-0075, Japan; Department of Orthopaedics, Juntendo University Faculty of Medicine, 2-1-1 Hongo, Bunkyo-ku, Tokyo 113-8421, Japan; Hada Medical Clinic, 1-46-20 Gotokuji, Setagaya-ku, Tokyo 154-0021, Japan; Department of Orthopaedics, Juntendo University Faculty of Medicine, 2-1-1 Hongo, Bunkyo-ku, Tokyo 113-8421, Japan; Department of Orthopaedic Surgery, Juntendo University, Tokyo Koto Geriatric Medical Center, 3-3-20 Shin-Suna, Koto-ku, Tokyo 136-0075, Japan; Department of Orthopaedics, Juntendo University Faculty of Medicine, 2-1-1 Hongo, Bunkyo-ku, Tokyo 113-8421, Japan

**Keywords:** nonunion, prolotherapy, bone healing, conservative treatment, biotherapy, pseudarthrosis, bone union, glucose, dextrose

## Abstract

Prolotherapy, involving hypertonic dextrose injections to stimulate growth factor release and promote tissue healing, has recently gained attention as a regenerative treatment for refractory musculoskeletal conditions. We report a 54-year-old man with a painful ulnar shaft nonunion after a karate injury. Conservative treatment failed over 12 months, and he presented with persistent pain (numeric rating scale [NRS] score 6). Ultrasound-guided prolotherapy using a 30% dextrose solution was performed in five sessions at 2–3 week intervals. Callus formation was noted at 4 weeks, pain completely resolved (NRS score 0), and the patient resumed karate at 12 weeks. Radiographic union was confirmed at 15 weeks without immobilization or surgery. This case highlights prolotherapy’s potential as a minimally invasive and effective alternative to surgery for painful nonunion.

## Introduction

Nonunion occurs in ~5%–10% of fractures and often poses a therapeutic challenge. Symptomatic cases typically require surgical intervention, which involves biological activation of the site through debridement and autologous bone grafting [1], along with re-stabilization using plates or intramedullary nails. However, these procedures are invasive and carry additional morbidity related to surgery and graft harvesting, with complication rates reported at 10%–30% [2]. In recent years, less invasive approaches for nonunion have gained attention, including therapies such as platelet-rich plasma derived from autologous blood [3, 4], extracorporeal shock wave therapy [5], and low-intensity pulsed ultrasound [6]. These modalities have shown promising results in promoting healing while minimizing surgical morbidity. Patients with nonunion often seek not only bone healing but also relief from chronic pain. Prolotherapy—local injection of hypertonic dextrose—has gained attention as a treatment for chronic musculoskeletal conditions involving bone, tendon, or ligament. This therapy is believed to promote tissue repair via growth factor release, enhance structural stability, and reduce pain by suppressing neovascularization involved in nociception [7].

We present a case of painful ulnar shaft nonunion successfully treated with dextrose prolotherapy. Nonunion was defined as failure to achieve radiological healing for ≥6 months with persistent symptoms.

## Case report

**Figure 1:**
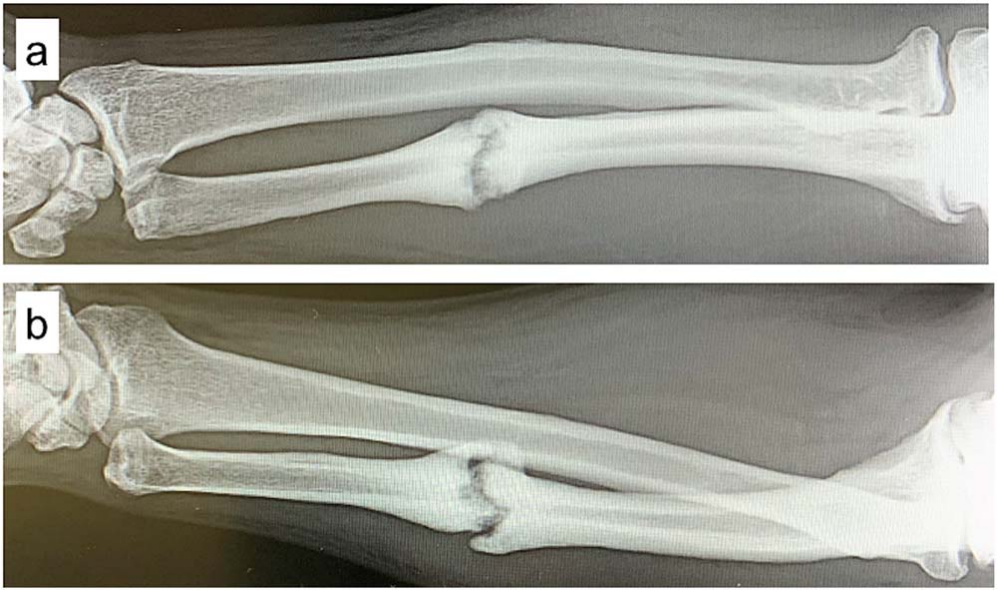
Plain radiographs at initial presentation. (a, b) Nonunion at the mid-diaphysis of the ulna is observed.

**Figure 2:**
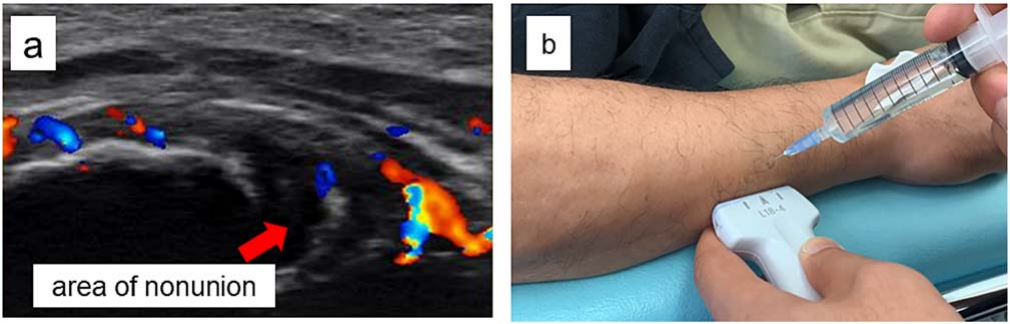
Ultrasound findings and images during the procedure. (a) Ultrasound image at the start of treatment showing marked Doppler signals around the nonunion site. (b) Dextrose injection into the nonunion site under ultrasound guidance.

**Figure 3:**
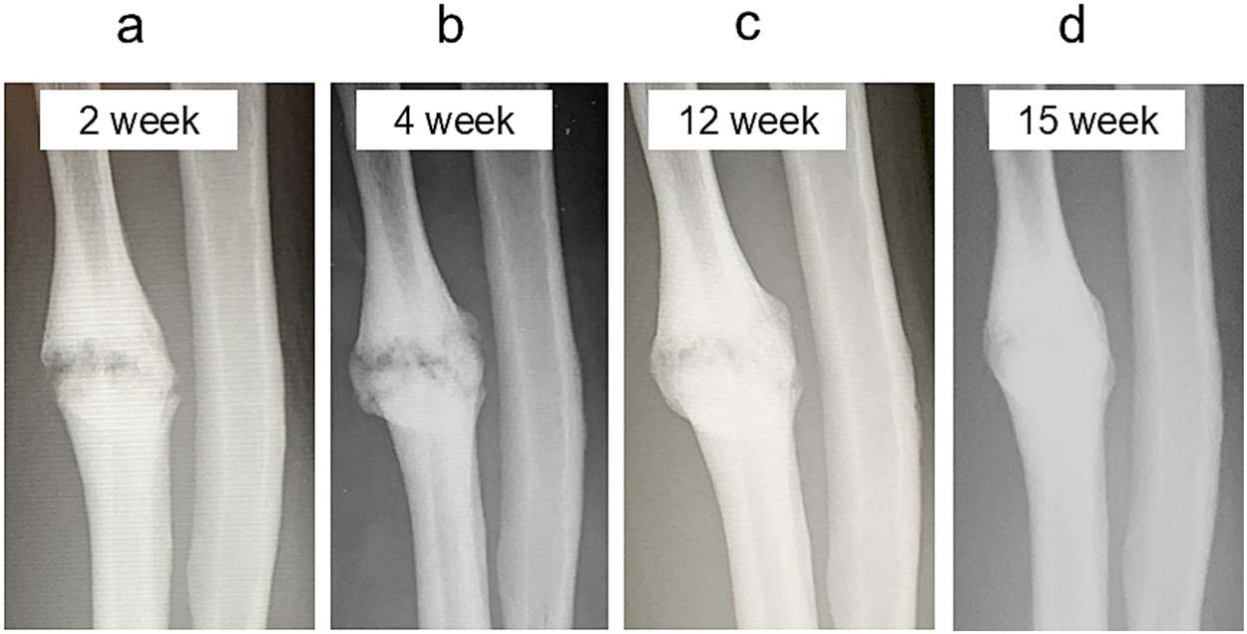
Radiographic findings during follow-up. (a) At 2 weeks, early callus formation is observed. (b) At 4 weeks, increased callus formation is noted. (c) At 12 weeks, near complete union is evident. (d) At 15 weeks, complete union achieved.

A 54-year-old man sustained a mid-diaphyseal ulnar fracture from a direct kick during karate practice. Initial conservative treatment at another hospital failed to achieve union over 12 months, and he was referred to our clinic with a diagnosis of nonunion. At presentation, he reported significant pain with a numerical rating scale (NRS) score of 6, and radiographs showed a 9 mm interfragmentary gap (Fig. 1a and b). Given his pain, we initiated proactive conservative management with prolotherapy, primarily targeting pain relief. Ultrasound revealed marked doppler signals at the nonunion site, suggesting inflammation and neovascularization. A mixture of 6 ml 50% dextrose and 4 ml 1% lidocaine (final 30% dextrose) was injected under ultrasound guidance into the neovascularized tissue and fracture gap (Fig. 2a and b). From the second session onward, bone healing had progressed to the point where the solution could no longer be injected between the bone fragments, so subsequent injections were administered around the periosteum. No restrictions were placed on weight-bearing or activity. Pain improved to NRS 4 at 2 weeks and to NRS 2 at 4 weeks, with early callus formation seen on radiographs. Prolotherapy was repeated five times at 2–3 week intervals. By 12 weeks, the patient was pain-free (NRS 0) and returned to karate. Radiographic union was confirmed at 15 weeks (Fig. 3a–d). Although initiated for pain control, prolotherapy led to relatively early bone union without surgery or complications.

## Discussion

For the treatment of refractory ulnar nonunion, the standard approach typically involves plate fixation combined with autologous bone grafting, with a reported average time to union of ~6.2 months [8]. In contrast, our case achieved bone union within 15 weeks after initiating treatment, without surgical intervention and without imposing any restrictions on daily activities, which represents a relatively early union. The possible mechanisms underlying the successful bone healing in this case may include the biological effects of prolotherapy. Prolotherapy is known to stimulate the release of growth factors, which could have exerted a secondary effect favoring bone union, as these growth factors are considered essential for nonunion healing [3]. Bone healing begins with hematoma formation, where fibrin and platelet-derived growth factors activate stem cells and osteoblasts, promoting extracellular matrix and hyaluronic acid synthesis. A lack

of these growth factors may lead to nonunion [9]. Osteoinductive molecules such as bone morphogenetic proteins and transforming growth factor-β (TGF-β) play key roles in nonunion healing [10]. Prolotherapy may have indirectly promoted their secretion, supporting bone repair. In addition, hypertonic dextrose could enhance bone formation by stimulating type I collagen synthesis and upregulating parathyroid hormone receptor expression, which may have contributed to the favorable outcome in this case [7, 11–13].

The optimal dextrose concentration for prolotherapy remains controversial, with studies reporting varying concentrations and effects [12]. The ideal dose likely depends on the specific pathology. For instance, a randomized controlled trial (RCT) for anterior cruciate ligament insufficiency showed significant improvement in knee stability with 10% intra-articular dextrose [13], while another RCT on Osgood-Schlatter disease found notable pain relief using 12.5% dextrose. Dextrose concentrations between 5% and 15% have also been associated with pain reduction, possibly through modulation of sensory nerve activity [7]. In contrast, higher doses like 50% have been used for pleurodesis in pneumothorax [14]. Overall, standardized, evidence-based concentration protocols are lacking and warrant further research.

Nonunion includes various subtypes with distinct etiologies. This case involved hypertrophic nonunion, characterized by preserved biological activity and healing potential. While spontaneous healing in nonunion has been reported in 5%–30% of cases [15], it is notable that union was achieved within 4 months after 12 months of failed healing—without immobilization or rest—suggesting a possible therapeutic effect of prolotherapy. The treatment used only dextrose and a local anesthetic, both widely available and inexpensive. It is minimally invasive, requires no surgery, and is relatively simple to perform. Although this is a single case, the early union without complications indicates that prolotherapy may serve as a promising conservative option for managing nonunion. Further controlled studies are needed to validate its efficacy.

## Conclusion

We report a case of symptomatic ulnar nonunion successfully treated with prolotherapy using hypertonic dextrose, resulting in bone union. Although this is a single case, the favorable outcome may support further exploration of prolotherapy as a conservative option for select cases of nonunion.
